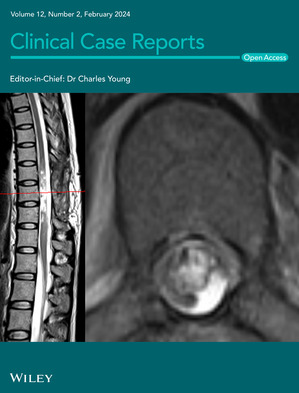# Cover Image

**DOI:** 10.1002/ccr3.8558

**Published:** 2024-03-07

**Authors:** Tadatsugu Morimoto, Hirohito Hirata, Takuya Nikaido, Kenichiro Taniguchi, Tomohito Yoshihara, Takaomi Kobayashi, Masatsugu Tsukamoto, Masaaki Mawatari

## Abstract

The cover image is based on the Case Report *Thoracic spinal epidural hematoma misdiagnosed as conversion paralysis: A case report* by Tadatsugu Morimoto *et al.*, https://doi.org/10.1002/ccr3.8434